# Multimodality Imaging and Management of a Giant Vertebral Artery Pseudoaneurysm: A Case Report and Review of Literature

**DOI:** 10.7759/cureus.82489

**Published:** 2025-04-18

**Authors:** Arvindh Sekaran, Pauline Gronczewska, Nathan Chan

**Affiliations:** 1 Internal Medicine, Addenbrooke's Hospital, Cambridge University Hospitals NHS Foundation Trust, Cambridge, GBR; 2 General Medicine, West Suffolk Hospital, West Suffolk NHS Foundation Trust, Bury St Edmunds, GBR; 3 Interventional Neuroradiology, Addenbrooke's Hospital, Cambridge University Hospitals NHS Foundation Trust, Cambridge, GBR

**Keywords:** balloon test occlusion, computed tomography angiography, magnetic resonance imaging, neurointerventional radiology, vertebral artery pseudoaneurysm, vessel sacrifice

## Abstract

Vertebral artery pseudoaneurysms (VAPAs) are rare but life-threatening lesions that present significant diagnostic and therapeutic challenges. Often resulting from trauma, infection, or iatrogenic injury, VAPAs can lead to severe morbidity due to their risk of rupture. This case report highlights a delayed diagnosis of VAPA in a male patient in his sixties presenting with recurrent falls, dysarthria, and bilateral limb paresthesia. Despite initial magnetic resonance imaging (MRI) findings suggestive of a nerve sheath tumor, subsequent computed tomography angiography (CTA) revealed a giant, partially thrombosed right VAPA. Advanced imaging techniques such as MRI and CTA were critical in accurately diagnosing this pseudoaneurysm. While MRI provided detailed soft tissue contrast, its interpretation was challenging. CTA offered clearer delineation of vascular structures, enabling better diagnostic accuracy and treatment planning. The patient underwent successful endovascular treatment with balloon test occlusion (BTO) and coiling, resulting in aneurysm occlusion while preserving the posterior circulation. Follow-up MRI, magnetic resonance angiography (MRA), and CTA confirmed stability post-procedure. This case underscores the importance of multimodal imaging for the accurate diagnosis of VAPAs. Additionally, it highlights the utility of endovascular approaches, including vessel sacrifice and coiling, in managing giant VAPAs. Tailored treatment strategies, based on vascular anatomy and collateral circulation, remain critical to optimizing patient outcomes.

## Introduction

Vertebral artery pseudoaneurysms (VAPAs) are rare but potentially life-threatening lesions that present significant diagnostic and therapeutic challenges. These pseudoaneurysms are typically acquired, often resulting from trauma, infection, or iatrogenic injury, and can develop in the extracranial segment of the vertebral artery due to its limited surrounding supportive tissue and the lack of protection provided by the skull base and dura mater [1]​​. The primary concern with VAPAs is their risk of rupture, which can lead to significant morbidity and mortality. As a result, accurate diagnosis and management are crucial, relying heavily on advanced imaging techniques such as magnetic resonance imaging (MRI) and computed tomography angiography (CTA) [2].

CTA is often utilized as a complementary imaging modality to MRI, providing high spatial resolution and rapid acquisition, which is particularly valuable in acute settings​. The detailed visualization of the vascular anatomy provided by CTA is crucial for both diagnosis and treatment planning. CTA offers high-resolution images that delineate the pseudoaneurysm from the parent artery with exceptional clarity. Additionally, three-dimensional reconstructions generated from CTA scans allow clinicians to fully understand the size, shape, and anatomical relationships of the lesion, which is essential for determining the best course of treatment​​. In scenarios where MRI findings are indeterminate, CTA can provide the necessary diagnostic confirmation and detailed anatomical information required for precise surgical or endovascular intervention [3].

Treatment options for VAPAs vary depending on the size, location, and cause of the pseudoaneurysm. Conservative management is often appropriate for small, stable, or mycotic pseudoaneurysms, particularly when patients have significant comorbidities [4]​​. However, in cases of symptomatic or enlarging pseudoaneurysms, more invasive treatments are required. Endovascular approaches, including the use of stents or the Pipeline Embolization Device (PED), have emerged as effective methods for treating these lesions by occluding the aneurysm while maintaining patency of the parent vessel​​ [5]. Additionally, in some cases, vessel sacrifice via balloon test occlusion (BTO) may be necessary if adequate collateral circulation can be confirmed​ [6].

This case report presents a delayed diagnosis of a VAPA and its consequent endovascular management, highlighting the importance of imaging modality and interpretation in the investigation of these lesions.

## Case presentation

A Caucasian right-hand dominant male in his sixties presented with a seven-month history of recurrent falls with dysarthria and bilateral upper and lower limb paresthesia and paresis, worse on the right-hand side. He had complained of up to 35 falls per month, with only being able to mobilize with a frame and not being able to traverse stairs, thereby effectively making him housebound in his first-floor flat. He was admitted to his local district general hospital (DGH), where standard blood tests revealed no abnormalities. They performed an MRI of his head that demonstrated a 7-cm mass posterior to the C1 vertebra at the craniocervical junction, with erosion of the skull base and compression of the adjacent cervicomedullary junction. The right vertebral artery was reported to be encased by this mass. This case was then referred to the regional skull base multidisciplinary team meeting, where a diagnosis of a nerve sheath tumor, possibly secondary to neurofibromatosis, was entertained. Upon review in the Neurosurgery clinic, the patient declined any operative management and was discharged to be managed by their general practitioner.

**Figure 1:**
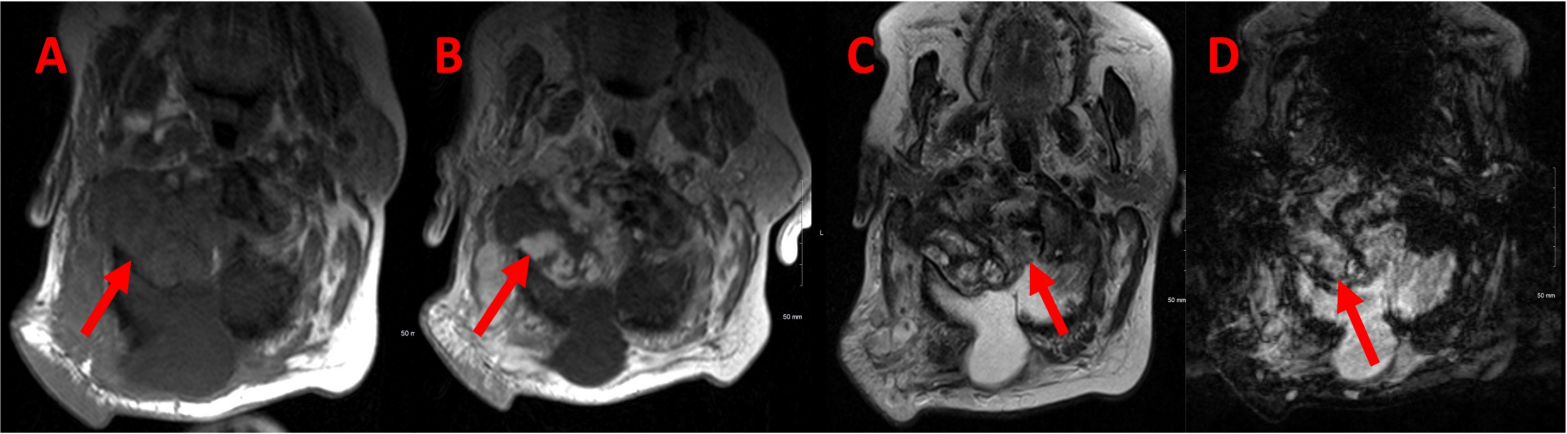
Axial magnetic resonance imaging of the patient's head - pre-treatment. (A) T1 (pre-contrast): The intermediate signal thrombus within the aneurysm is relatively innocuous on T1 (red arrow). (B) T1 (post-contrast): Post-contrast images demonstrate central mass-like enhancement of the aneurysm (red arrow). (C) T2: Mixed signal within the aneurysm, which displaces and compresses the cervicomedullary junction (red arrow) to the left. (D) Susceptibility weighted imaging (SWI): Susceptibility artifact is seen outlining the aneurysm wall (red arrow), another clue to the vascular nature of the pathology.

**Figure 2:**
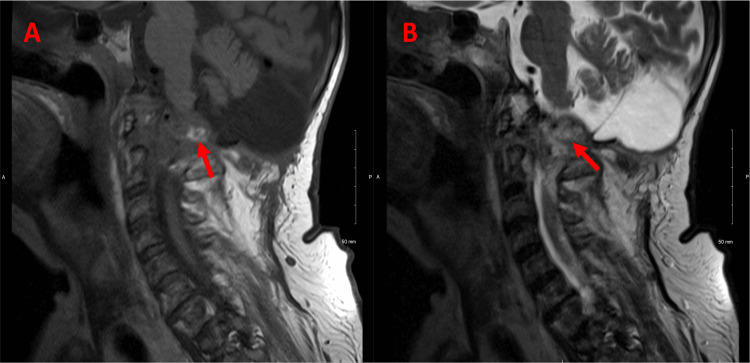
Sagittal magnetic resonance imaging of the patient's head - pre-treatment. (A) T1: Small areas of T1 high-signal thrombus (red arrow) are noted within the aneurysm on the sagittal images. (B) T2: The aneurysm is located at the craniocervical junction and erodes the posterior arch of the atlas and squamous portion of the occipital bone (red arrow).

**Figure 3:**
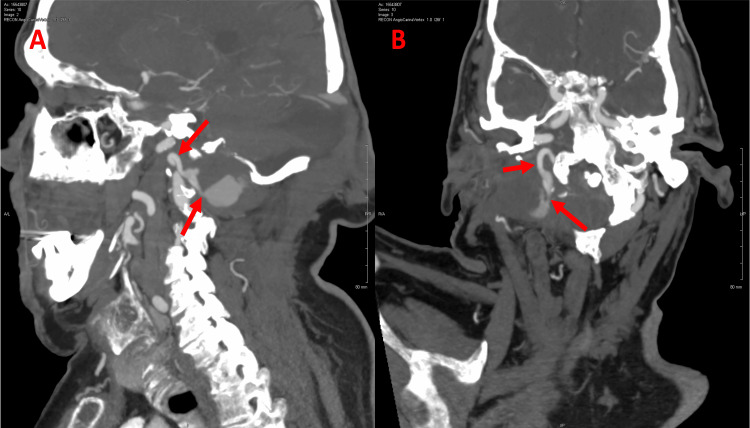
Computed tomography angiography of the patient's head and neck arteries - pre-treatment. (A) Sagittal section; (B) axial section. Communication between the right vertebral artery and the mass with contrast material leaking from the vessel to the lesion (red arrows).

Over the following month, he experienced worsening headaches and a decline in mobility, which led to his placement in a nursing home and subsequent readmission to his DGH. A repeat MRI of the head demonstrated an increase in the size of the known mass, with further displacement of the spinal cord to the left (Figures 1, 2). The right vertebral artery was deemed to be compressed by this mass, as it was difficult to visualize on the MRI. The patient then opted for surgical intervention, requiring elective admission under the Neurosurgical team at our tertiary care hospital. Neurosurgery advised to perform a CTA of the aortic arch and bilateral carotid arteries to visualize the vertebral arteries to help plan surgical intervention. This CTA demonstrated that the condylar and squamous component of the occipital bone showed marked remodeling with cephalad bowing, suggesting a longstanding/slow-evolving lesion. The mass showed heterogeneous density, mostly hypodense but with a large hyperdense component with density mildly lower than the adjacent vertebral artery crossing anteriorly to the lesion itself. The enhancing component of the mass communicated with the right vertebral artery, indicating a giant partially thrombosed pseudoaneurysm of this vessel (Figure 3). The distal V2 and V3 segments of the right vertebral artery appeared irregular in caliber with segments of fusiform dilatation.

**Figure 4:**
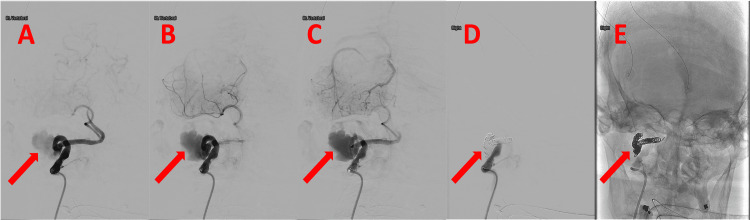
Digital subtraction angiography of the patient's head and neck vessels - anterior-posterior projection. (A-C) Pre-treatment (subtracted): The red arrows indicate the location of the pseudoaneurysm. (D) Post-treatment (subtracted): The red arrow indicates the location of vessel coiling. (E) Post-treatment (unsubtracted): The red arrow indicates the location of vessel coiling.

**Figure 5:**
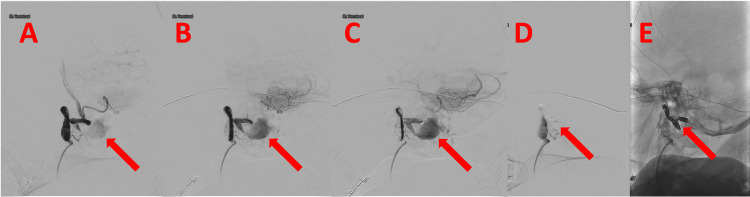
Digital subtraction angiography of the patient's head and neck vessels - lateral projection. (A-C) Pre-treatment (subtracted): The red arrows indicate the location of the pseudoaneurysm. (D) Post-treatment (subtracted): The red arrow indicates the location of vessel coiling. (E) Post-treatment (unsubtracted): The red arrow indicates the location of vessel coiling.

**Figure 6:**
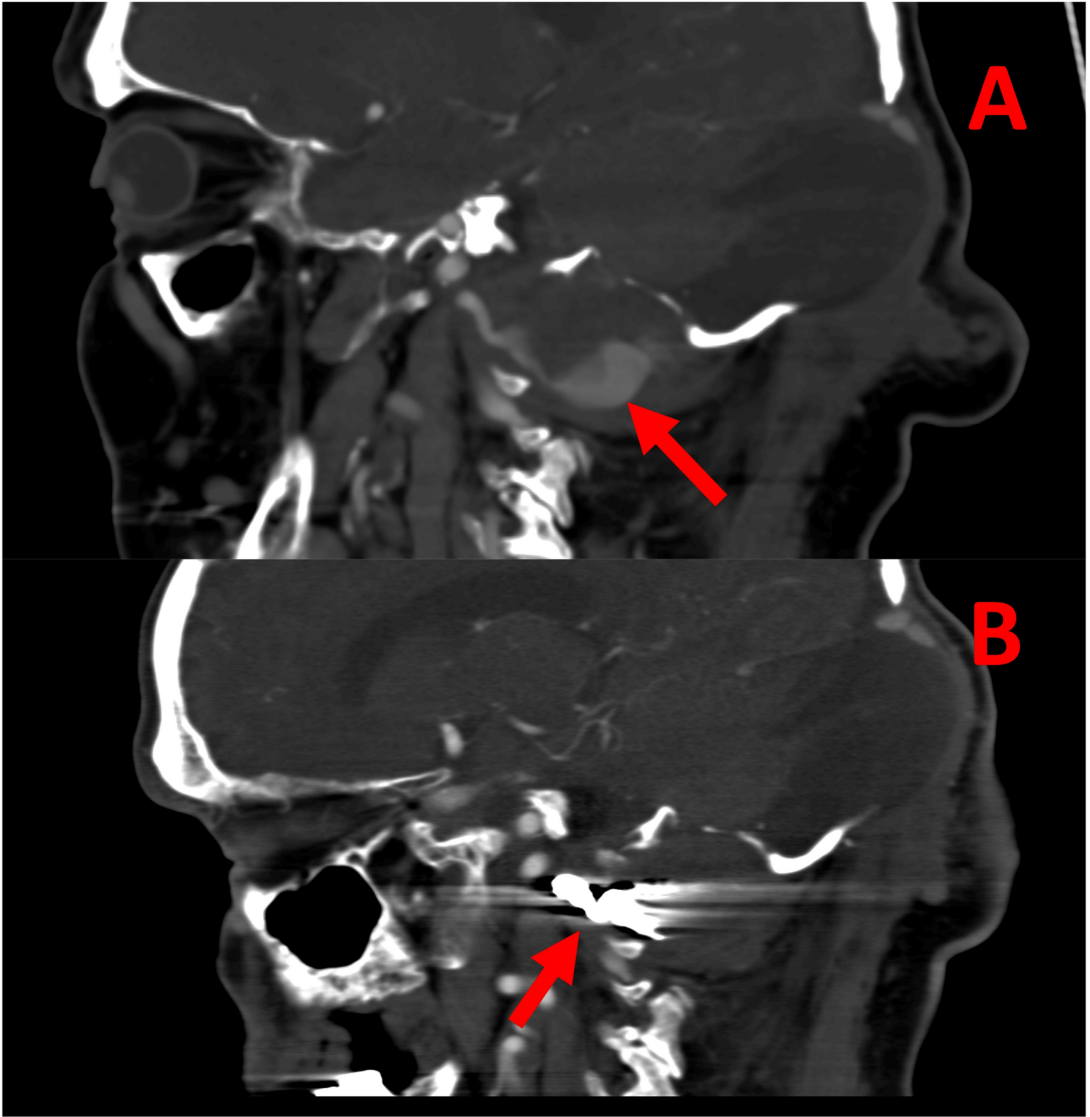
Computed tomography angiography of the patient's right vertebral pseudoaneurysm - pre-treatment and 11 months post-treatment. (A) Pre-treatment sagittal section: Contrast leaking into the culprit pseudoaneurysm from the right vertebral artery (red arrow). (B) Post-treatment sagittal section: Coils are present in the pseudoaneurysm neck and proximal right vertebral artery (red arrow) and no filling is seen in the pseudoaneurysm.

The CTA was reviewed by the Neurointerventional Radiology team, who identified the pseudoaneurysm before the formal radiology report. A plan was made to perform a digital subtraction angiography (DSA) with BTO of the right vertebral artery under local anesthetic, with immediate definitive treatment in the form of vessel sacrifice if the BTO was tolerated. Flow diversion and coiling were a possible alternative if the patient failed BTO; however, it was felt that this would be suboptimal due to the risks of stent compression secondary to movement at the craniocervical junction. DSA confirmed a giant partially thrombosed right VAPA with the neck of the aneurysm arising from the V3 segment, directed inferiorly (Figures 4, 5). The patient tolerated BTO for 25 minutes after the balloon was advanced into the right V2 segment and inflated. Multiple detachable coils were placed into the parent vessel distal to the aneurysm, within the filling component of the aneurysm, and proximal to the aneurysm, to the level of the C1 lateral mass. Angiography via the left vertebral artery at the end of the procedure confirmed occlusion of the aneurysm with normal filling of the posterior circulation. A three-month follow-up MRI with magnetic resonance angiography (MRA) showed occlusion of the pseudoaneurysm, but there was too much motion blur to interpret it further; hence, another CTA was performed 11 months post-treatment (Figure 6). This showed the VAPA remained occluded with stable appearances. The patient was reviewed in clinic 12 months post-treatment, with his neurological symptoms having remained stable; hence, he was discharged.

## Discussion

**Figure 7:**
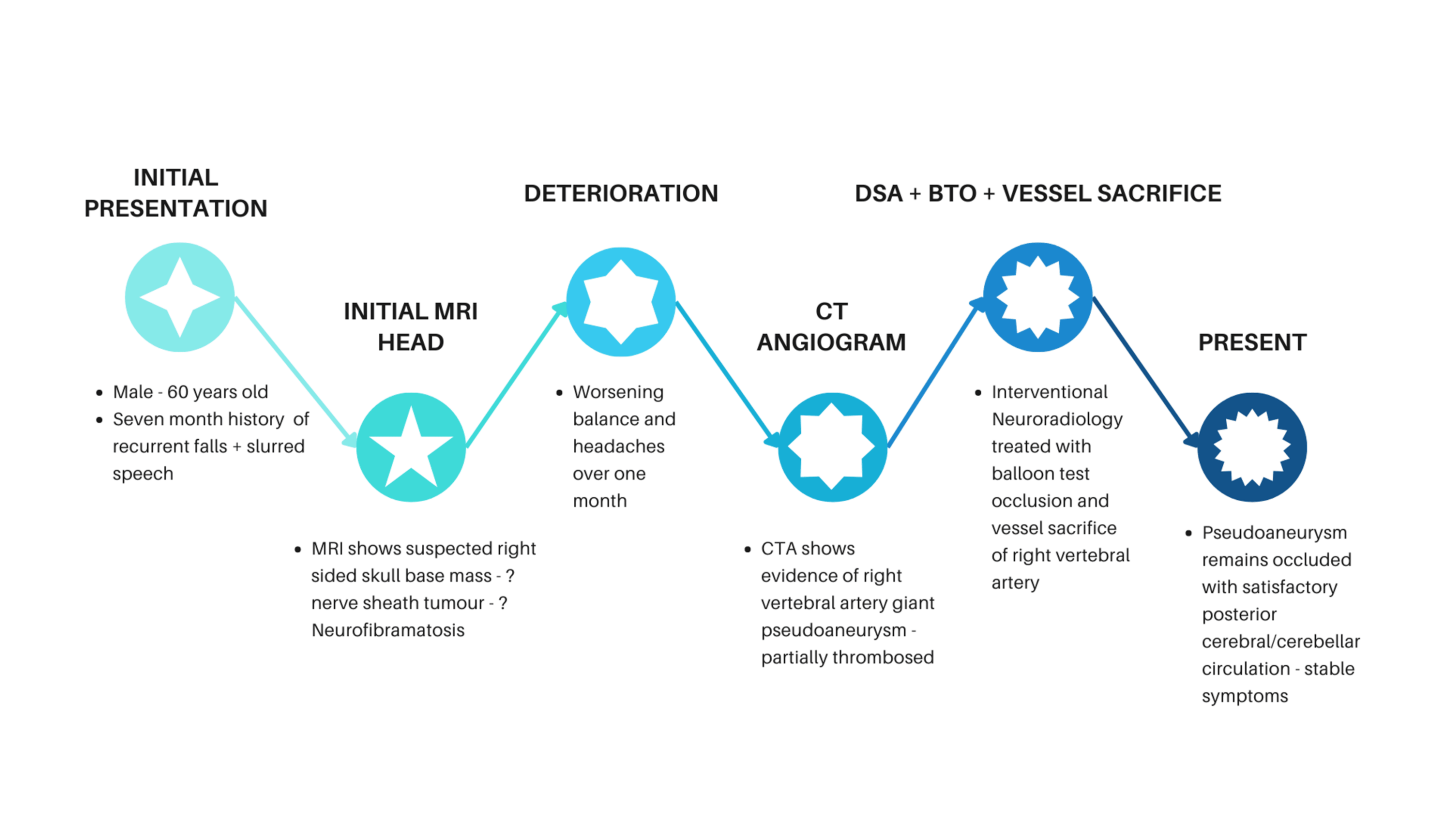
Overall timeline of the patient's journey. Image credit: Arvindh Sekaran. MRI, magnetic resonance imaging; CTA, computed tomography angiography; DSA, digital subtraction angiography; BTO, balloon test occlusion

This case demonstrates the successful endovascular treatment of a giant VAPA with BTO and vessel sacrifice through coiling (Figure 7). Although the mass was initially thought to be a nerve-sheath tumor with mass effect, this can be attributed to features on images obtained during MRI, which can lead to false negatives, as outlined by Provenzale et al. [7].

MRI plays a vital role in the evaluation of VAPAs, primarily due to its excellent soft tissue contrast and ability to detect subtle changes in vascular and surrounding tissue. Several key findings on MRI suggest the presence of a pseudoaneurysm. One of the most critical diagnostic clues is the presence of low or mixed T2 signal, which may represent thrombus and therefore raises suspicion of a vascular pathology​​. Additionally, irregular luminal flow voids within a vertebral artery suggest abnormal blood flow, characteristic of vascular lesions like pseudoaneurysms. Also in our case, the pseudoaneurysm, at the time thought to be a mass, did not demonstrate diffusion restriction, which may be seen in more aggressive lesions such as tumors. Finally, the mass effect, which is nonspecific but significant, demonstrates the compression of adjacent structures by the growing pseudoaneurysm​. These findings are essential for raising suspicion of a VAPA, but given the complexity of the surrounding anatomy, MRI alone may not provide a definitive diagnosis, especially in cases with ambiguous findings [8]. For example, on T1-weighted images, the intramural hematoma of an intracranial artery dissection is often the same signal intensity as surrounding tissues, and any resulting pseudoaneurysms with signal intensities reflecting abnormal flow are often mistaken for a mass.

On the contrary, CTA offers higher sensitivity in detecting vertebral artery dissections and resultant pseudoaneurysms, with better availability and tolerability being crucial considerations when offering this type of imaging [2]. Obtaining a CTA in this case was helpful and serves as a reminder that if there is clinical doubt of MRI appearances at the skull base and a vascular injury/anomaly has not been ruled out, then a CTA is a quick and easy tool to use for this purpose.

As for the treatment of VAPAs, this must be a tailored approach to decide between conservative, endovascular, and surgical treatments. Historically, if the lesion was accessible, then surgical treatment was employed, and if not, then conservative treatment with antiplatelet/anticoagulants was the mainstay [9]. Endovascular strategies that have been implemented for pseudoaneurysms include covered stents, flow diverting stents, BTO, and vessel sacrifice with preservation of the ipsilateral posterior inferior cerebellar artery (PICA) [3,6,10-14]. The latter approach is not feasible if the dominant vertebral artery is affected or the contralateral vascular supply is insufficient or hypoplastic. Anatomical variations will also play a role in choosing appropriate management. For example, PICA normally originates from the V4 segment but is known to have variable origins, including the V3 segment, which is known to be the most vulnerable to injury and pseudoaneurysm formation due to its extradural location and lack of structural protection of the transverse foramen. If the origin of PICA is compromised by vessel sacrifice or stents, then this in turn will significantly impact the posterior cerebral/cerebellar circulation [15].

## Conclusions

In conclusion, VAPAs are rare but life-threatening vascular lesions that require a high index of suspicion and multimodal imaging for accurate diagnosis and effective management. As demonstrated in this case, MRI, while valuable for soft tissue evaluation, may yield ambiguous findings, emphasizing the role of CTA in providing clearer vascular delineation. Endovascular techniques, particularly vessel sacrifice following BTO, offer a viable and less invasive treatment option for managing such aneurysms, preserving neurological function and preventing rupture. Tailored treatment strategies, based on vascular anatomy and collateral circulation, are essential for optimizing outcomes.

## References

[1] Schittek A (1999). Pseudoaneurysm of the vertebral artery. Tex Heart Inst J.

[2] Gottesman RF, Sharma P, Robinson KA, Arnan M, Tsui M, Saber-Tehrani A, Newman-Toker DE (2012). Imaging characteristics of symptomatic vertebral artery dissection: a systematic review. Neurologist.

[3] Carrillo-Martínez MÁ, Garza García GA, Leal Jacinto JM (2020). Iatrogenic left vertebral artery pseudoaneurysm treated with a covered stent. BJR Case Rep.

[4] Ducruet AF, Hickman ZL, Zacharia BE, Narula R, Grobelny BT, Gorski J, Connolly ES Jr (2010). Intracranial infectious aneurysms: a comprehensive review. Neurosurg Rev.

[5] Zhang H, Zhang H, Liu J (2023). Pipeline embolization device for small and medium vertebral artery aneurysms: a multicenter study. Neurosurgery.

[6] Zoarski GH, Seth R (2014). Safety of unilateral endovascular occlusion of the cervical segment of the vertebral artery without antecedent balloon test occlusion. AJNR Am J Neuroradiol.

[7] Provenzale JM, Sarikaya B, Hacein-Bey L, Wintermark M (2011). Causes of misinterpretation of cross-sectional imaging studies for dissection of the craniocervical arteries. AJR Am J Roentgenol.

[8] Zhang M, Ye G, Liu Y, Wang Q, Li S, Wang Y (2019). Clinical application of high-resolution MRI in combination with digital subtraction angiography in the diagnosis of vertebrobasilar artery dissecting aneurysm: an observational study (STROBE compliant). Medicine (Baltimore).

[9] Larson PS, Reisner A, Morassutti DJ, Abdulhadi B, Harpring JE (2000). Traumatic intracranial aneurysms. Neurosurg Focus.

[10] Ambekar S, Sharma M, Smith D, Cuellar H (2014). Successful treatment of iatrogenic vertebral pseudoaneurysm using pipeline embolization device. Case Rep Vasc Med.

[11] Méndez JC, González-Llanos F (2005). Endovascular treatment of a vertebral artery pseudoaneurysm following posterior C1-C2 transarticular screw fixation. Cardiovasc Intervent Radiol.

[12] Koueik J, Larson S, Ahmed A, Hanna AS (2023). Large vertebral artery pseudoaneurysm masquerading as a schwannoma: illustrative case. J Neurosurg Case Lessons.

[13] Cohen JE, Gomori JM, Rajz G, Rosenthal G, El Hassan HA, Moscovici S, Itshayek E (2016). Vertebral artery pseudoaneurysms secondary to blunt trauma: endovascular management by means of neurostents and flow diverters. J Clin Neurosci.

[14] Madaelil TP, Wallace AN, Chatterjee AN (2016). Endovascular parent vessel sacrifice in ruptured dissecting vertebral and posterior inferior cerebellar artery aneurysms: clinical outcomes and review of the literature. J Neurointerv Surg.

[15] Miao HL, Zhang DY, Wang T, Jiao XT, Jiao LQ (2020). Clinical importance of the posterior inferior cerebellar artery: a review of the literature. Int J Med Sci.

